# Mandibular Bone and Soft Tissues Necrosis Caused by an Arsenical Endodontic Preparation Treated with Piezoelectric Device

**DOI:** 10.1155/2013/723753

**Published:** 2013-08-22

**Authors:** A. Giudice, M. G. Cristofaro, I. Barca, D. Novembre, M. Giudice

**Affiliations:** ^1^Department of Oral and Maxillofacial Surgery, University Magna Graecia, 88100 Catanzaro, Italy; ^2^University Magna Graecia, 88100 Catanzaro, Italy; ^3^Maxillofacial Unit, University Magna Graecia, 88100 Catanzaro, Italy

## Abstract

This paper describes a case of wide mandibular bone necrosis associated with significant soft tissues injury after using an arsenical endodontic preparation in the right lower second molar for endodontic purpose. Authors debate about the hazardous effects of the arsenic paste and the usefulness of piezosurgery for treatment of this drug related bone necrosis.

## 1. Introduction

Arsenic paste was used to devitalise dental pulp in particular cases where endodontic surgical measures (e.g., vital extirpation) were not possible. Its use in contemporary dentistry should be avoided, because it no longer has a therapeutic role and has been proven to have many hazardous effects. In fact, arsenic and its compounds may produce severe damage of the periapical tissue, destruction of supporting bone, and the loss of teeth when left in the pulp chamber for a long period of time [1–3].

Another disadvantage of using arsenicals is the difficulty in limiting their uncontrolled spread and their non-self-limiting action [4].

## 2. Case Report 

A 48-year-old woman presented to the maxillofacial unit of Università Magna Graecia di Catanzaro in July 2012 with severe pain in the right jaw associated with limited mouth opening.

**Figure 1:**
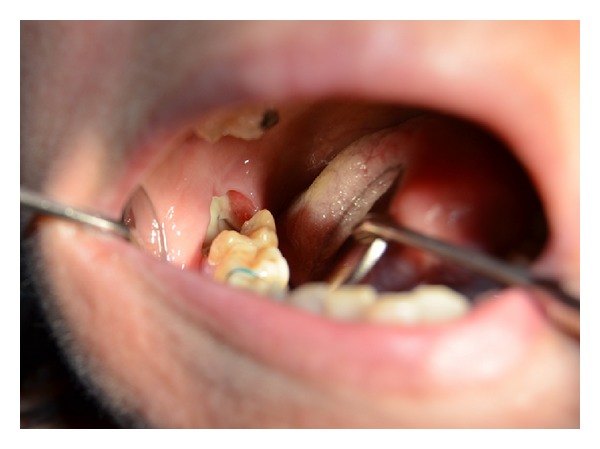
Clinical view of the area of necrosis and inflammation in maxillary second and third molars.

**Figure 2:**
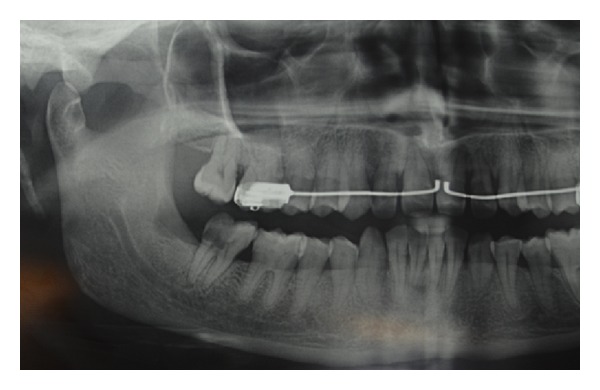
Preoperative radiographic appearance of radiolucent area back to tooth 47.

**Figure 3:**
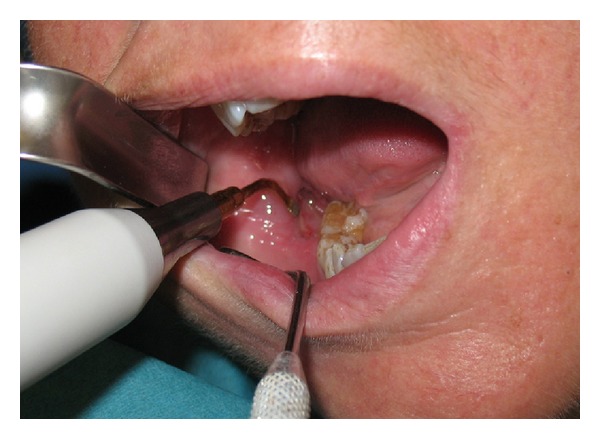
Intraoral view of sequestrectomy.

**Figure 4:**
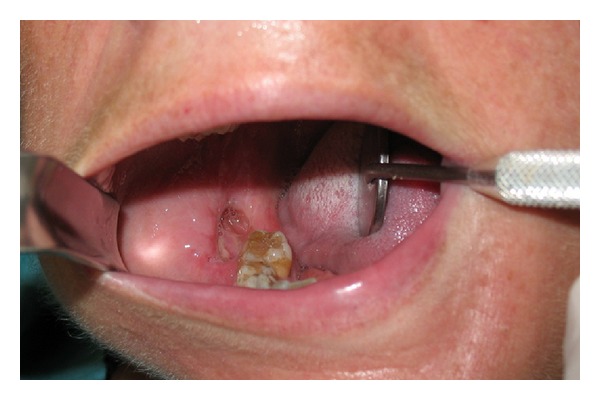
Clinical healing after surgical treatment.

**Figure 5:**
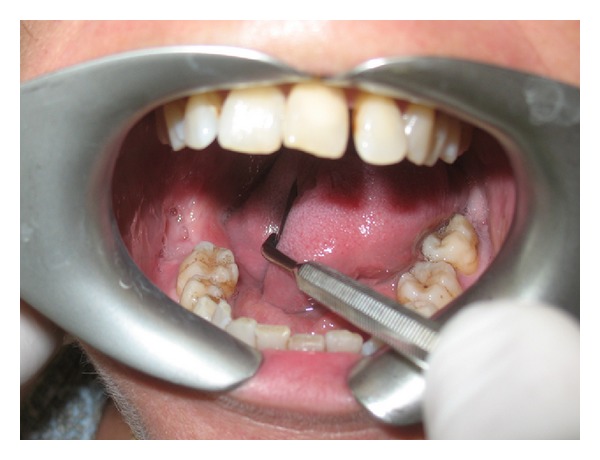
Three months clinical follow-up showed complete healing of the molar area.

**Figure 6:**
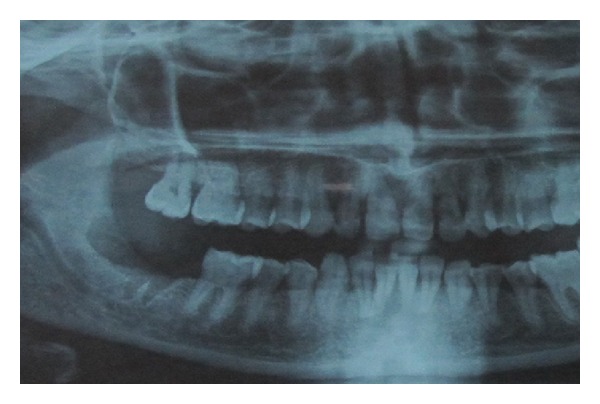
Radiographic appearance of persistent bony lucency in third molar and retromolar area after three months.

At the dental history, the patient referred about an endodontic treatment that was performed 15 days before; she referred, the day after the treatment, during the meal, the loose of provisional restoration. She experienced in the next three days later a worsening and severe pain of the area. Referring to her GD, she was treated with antibiotics and steroids. Three days later the patient's complaints did not improve and the GD recommended a specialist consultation. At clinical examination, the oral cavity showed an extensive (2.5 × 1.5 cm) area of bone aseptic necrosis regarding the right lower molar and retromolar area with a wide inflammation of the surrounding buccal mucosa (Figure 1). Tooth 47 presented without any temporary restoration. Panoramic radiography revealed a radiolucent area back to tooth 47 (Figure 2). On clinical and radiological findings, chemical aseptic necrosis of the mandible was diagnosed. An interview with her general dentist confirmed the use of an arsenical paste medication during root canal treatment of 47. The area was surgically treated under local anesthesia performing tooth 47 removal, sequestrectomy of bone, and soft tissue debridement (Figure 3). Sequestrectomy was made using a piezoelectric cut. Primary closure of the wound was secured with a 3–0 absorbable suture. A course of oral amoxicillin trihydrate 825 mg plus potassium clavulanate 125 mg was given twice a day for 10 days. Additionally, the patient received oral ketoprofen lysine salt 80 mg as an analgesic and dexamethasone 25 mg as an anti-inflammatory agent both once a day for 5 days and chlorhexidine gluconate mouthwash two times a day for 5 days as a local antiseptic. A soft diet was advised during the first postoperative week, and the patient was recalled after 5 days to evaluate healing. The clinical examination confirmed a painless healing immediately after the sequestrectomy (Figure 4). At the three months clinical followup (Figure 5), the panoramic radiography (Figure 6) showed the complete healing of the right lower retromolar region.

## 3. Discussion

Osteonecrosis is a severe bone disorder, traditionally associated with different diseases as periodontal, traumas and malignancies. Today bisphosphonates are the commonest drugs associated with a direct toxicity on osteocytes [5]. In the past, other chemotherapeutic agents as arsenic trioxide and paraformaldehyde were associated with bone necrosis. Once, these drugs were commonly employed as pulp-necrotizing agents.

The first use of arsenic trioxide in medicine as a devitalizing agent dates back to 994 AC with Haly Abbas and subsequently in 1836 with Spooner [6, 7]. Its cytotoxic effects are well recognized, and the leakage from the teeth to the surrounding tissue has been associated with widespread necrosis of the periodontal tissue and bone [8–10]. Arsenic paste was once also used to devitalize inflamed pulp tissues. Despite its hazardous effects, it is used in strictly controlled doses in the treatment of diseases such as solid tumours, multiple myeloma, and acute promyelocytic leukaemia but not for endodontic purposes. The looseness of arsenical paste from the pulp chamber can cause a severe soft tissues necrosis and osteomyelitis [11, 12]. The first case of complication due to the use of arsenic compound in endodontic reported in the literature dated back to 1957 [13]. From that date, 18 cases of arsenical paste-related bone necrosis have been reported [1, 2, 4, 7–16]. Maxilla and mandible have the same frequency of occurrence. Both, bone and soft tissue necroses were observed in all cases. Dental practitioners should be aware of using arsenical paste as devitalizing agent: its diffusion into periodontal and surrounding tissues through dental canals, perforations, or, as in the case reported, the leaking of the temporary restoration can lead to severe complications [14–17]. Depending on the severity of the case, the treatment can be conservative or surgical. The conservative treatment is a combination of maintenance therapies such as endodontic, periodontal, and pharmacological observations. Often both pharmacotherapeutic and invasive surgical therapies are required. In many cases, tooth extraction and sequestrectomy as the excision of all necrotic surrounding soft tissue may be necessary. After such surgical treatment, the loss of the alveolar bone and attached gingiva is unavoidable. To minimize the side effects of nonconservative therapy, authors propose a piezoelectric assisted sequestrectomy. Piezosurgery, thanks to fine modulating ultrasonic microvibrations, is able to cut the mineralized tessue respecting soft tissues. Experimental and clinical applications clearly show the decrease of the risk in damaging soft tissues and critical structures (nerves, vessels, and mucosa). Piezoelectric device seems to have a biostimulating effect on the surrounding tissues, inducing neoangiogenesis, proliferation of epithelial tissue, and increasing the osteogenesis [18, 19]. Piezoelectric bone surgery seems to be more efficient in the first phases of bony healing; it induces an earlier increase in bone morphogenetic proteins, controls the inflammatory process better, and stimulates remodelling of the bone [20, 21]. Its physical and mechanical properties have several clinical advantages, especially in such difficult cases; the precise cutting, the sparing of vital neurovascular bundles, the better visualization of the surgical field, and the limited amount of local anaesthesia needed are beneficial for the surgeon. 

## 4. Conclusion

Arsenic compounds cause severe consequence and should not be used in endodontic practice. They have no more place in dental practice, and practitioners should employ local anesthetics to achieve pulp extirpation. Often dental extraction and sequestrectomy are mandatory. Osseous surgery was made thanks to special properties of piezoelectric cut. This technique was particularly beneficial for patient with bone necrosis. The surgical procedure can be performed in a safer way and under local anesthesia.
